# Pharmacy preparedness and response for the prevention and control of coronavirus disease (COVID-19) in Aksum, Ethiopia; a qualitative exploration

**DOI:** 10.1186/s12913-020-05763-9

**Published:** 2020-10-02

**Authors:** Gebremicheal Gebreslassie Kasahun, Gebremichael Mulu Kahsay, Amha Teklu Asayehegn, Gebre Teklemariam Demoz, Desilu Mahari Desta, Gebremedhin Beedemariam Gebretekle

**Affiliations:** 1 Department of Pharmacy, College of Health Sciences, Aksum University, Aksum, Tigray Ethiopia; 2 School of Pharmacy, College of Health Sciences, Mekelle University, Mekelle, Tigray Ethiopia; 3 School of Pharmacy, College of Health Sciences, Addis Ababa University, Addis Ababa, Ethiopia

**Keywords:** Pharmacy service, Community pharmacy, Hospital pharmacy, COVID-19, SARS-CoV-2

## Abstract

**Background:**

Novel Coronavirus is a global pandemic affecting all walks of life and it significantly changed the health system practices. Pharmacists are at the front line and have long been involved in combating this public health emergency. Therefore, the study was aimed to explore pharmacy preparedness and response to prevent and control coronavirus disease 2019 (COVID-19).

**Methods:**

A qualitative study was conducted in six pharmacies in Aksum, Ethiopia in May, 2020. We conducted six in-depth interviews with purposively selected key informants. Direct observation measures were made to assess the activities made in the medicine retail outlets for the prevention and control of the pandemic. Interview data were audio-recorded, translated and transcribed verbatim. Thematic analysis was employed to analyze the data and OpenCode version 4.02 software was used to facilitate the data analysis.

**Results:**

The thematic analysis has resulted in seven major themes. Good preparedness measures were undertaken to control and prevent COVID-19. Study informants had good knowledge about the pandemic disease and reported they had used different resource materials to update themselves. Preparing of alcohol-based hand-rub, availing finished sanitizers and alcohol, and advising clients to maintain physical distancing were the major counseling information being delivered to prevent the disease. Some tendencies of irrational drug use and false claims of COVID-19 were observed at the beginning of the pandemic. Interview informants had reported they were working with relevant stakeholders and appropriate patient education and support were given to combat the pandemic.

**Conclusion:**

The study revealed necessary pharmacy services has been rendered to all clients. However, availability of drugs and medical supplies were scarce which negatively affected the optimal delivery of pharmacy services. The government and other responsible bodies should work together to solve such problems and contain the pandemic.

## Background

A novel coronavirus is currently an outbreak of respiratory disease worldwide that is caused by severe acute respiratory syndrome-coronavirus-2. The virus causes Coronavirus Disease 2019 (COVID-19) which has been recently declared as global pandemic by WHO on March 11, 2020 [1, 2]. Currently, COVID-19 is found to be one of the most contagious and virulent viruses that challenged the globe [3]. Thus, COVID-19 has conceived the foremost to spread to the entire globe and brought substantial health crisis and keeps to pose a global threat [4].

Community pharmacists are vital healthcare providers during the outbreak by serving as direct points of access for their patients and on the frontline of public health. Likewise, Hospital pharmacists have an expert role in providing evidence based pharmaceutical care service and managing the drug supply system which ultimately optimize the infection control as well as patient care and support [5]. The community pharmacists continue to play their role in providing information on precautions related to COVID-19 spread. Thus, community and hospital pharmacists provide essential services to patients and healthcare teams through continued provision and supply of medicines and treatment [2, 6]. The Ethiopian ministry of health has issued standard operating procedure for preparation of hand sanitizers for compounding pharmacists and manufacturers to cope with the stock outs [7]. In relation with drug supply chain, it is left vulnerable and this in turn poses burdens on healthcare providers and the entire healthcare system [8]. In Low- and Middle-Income Countries (LMICs), movement restrictions, is considered as a primary mitigation strategy to prevent COVID-19 transmission. Self-isolation, physical distancing, shielding of high-risk individuals should be extended until a treatment and vaccine options become available [9]. Pandemics cause significant morbidity and mortality impacts particularly in LMICs. As a result, the prevention strategies also result unintended social and economic disruption [10].

The role and responsibilities of pharmacists needs to be explored in the context of COVID-19 pandemic thereby reducing the health service burden. It is also important to profoundly assess pharmacists’ readiness and full potential use is implemented to prevent the pandemic. Thus, the study explored the pharmacy service preparedness and response to prevent and control COVID-19.

## Methods

### Study area and period

The study was carried out in Aksum city, Tigray Regional State, Northern Ethiopia. A total of sixteen private medicine retail outlets (four pharmacies, eleven drug stores and one drug vendor) are found in the city. In addition, four governmental healthcare facilities; one comprehensive specialized hospital, one general hospital and two health centers are found in the city. The study was conducted in May, 2020.

### Study design

A qualitative study was employed to explore pharmacy service preparedness undertaken to fight COVID-19 in hospital and community pharmacies. In-depth interview and direct observation were made using a semi-structured data collection instrument developed based on literatures pertinent to our study [11, 12] to assess the preparedness of pharmacies and pharmacy staffs to combat COVID-19.

### Data collection

Data was collected from medicine retail outlets found in Aksum city which were first listed down consulted from Aksum Town Health Office. Community pharmacies and hospital pharmacies which share major pharmacy service in the city were the targeted study areas. In-depth interview was made with pharmacy heads using a semi-structured interview guide comprised of probing questions. Study informants were selected purposively and interview was stopped in the sixth interview due to saturation of information. The interview guide was initially prepared in English language which later translated to Tigrigna (local language) and back-translated to English to ensure consistency. Pretesting was carried out in two medicine dispensaries and appropriate modification was made in the final version of the interview guide. The interview guide developed for this study is provided as Additional file 1 (Supplementary materials).

The data collection instrument consisted questions about socio-demographic information, pharmacy preparedness activities against COVID-19, patient education and support given for clients and assessment tips checked on direct observation. Two trained pharmacists collected the data. The average duration of interview was 30 min, ranged from 25 to 35 min. All interviews were made in Tigrigna language which was audio-recorded. In addition, direct observation was made to assess the preparedness activities being carried out against COVID-19 which helped us in triangulating with the finding of the in-depth interview.

### Data management and analysis

Data were coded using single-blinded identification code. Audio-recorded interview data were transcribed which later translated to English language. This was made by bilingual authors GGK and ATA. This transcribed verbatim which later translated and the observational checklist obtained notes were carefully read and categorized in to seven major themes emerged during our thematic analysis. All co-authors of the study verified the content of the themes created. OpenCode version 4.02 software was used to facilitate the analysis of the data.

## Results

A total of six pharmacists were interviewed. Majority (*n* = 5) of the study informants were males with an average age of 35.6 years ranged from 27 to 53 years. All study informants had bachelor’s degree in pharmacy and on average 12 years of work experience ranged from 2 to 30 years. Four pharmacists were working in community pharmacies and were owners of the pharmacy. The remaining two pharmacists were working in hospitals as a case team coordinator (Table 1).

**Table 1:** Socio-demographic characteristics of study informants

Variables	Frequency (N)
Age (in years)	
21–30	3
31–40	1
> 40	2
Gender	
Male	5
Female	1
Level of education	
BPharm (degree)	5
M.Sc. and above	1
Experience (in years)	
1–5	2
6–10	2
> 10	2
Premise areas pharmacy service provided	
Community pharmacy	4
Hospital pharmacy	2

### Awareness about COVID-19

All (*n* = 6) pharmacists responded correctly to COVID-19 history, transmission and prevention strategies. The study showed that all respondents were aware of the disease and they had up-to-dated information about the pandemic. Different resources such internet, web pages and social-medias were mentioned as the main source of knowledge to update the recent development and progress of the disease. One of the respondents stated that:

*“…COVID-19 is a disease caused by to a new strain of coronavirus family that affects the respiratory system. It was firstly reported from Wuhan, China in December 2019 and it became a pandemic disease affecting populations worldwide. The main transmission mechanisms are droplets, contact and airborne. An individual who has got the disease can transmit the disease while sneezing, handshaking and through surface contact. Therefore, it can be easily prevented by isolating suspected individuals, practicing physical distancing, and hand washing using soap and water for 20-30 seconds or sanitizing our hands. Drugs and vaccines are under development. I always refer to WHO guideline and web page when I want to update my knowledge about the case.” (C19Phar002)*

### Preparedness to combat the pandemic disease

During the study period, all informants (*n* = 6) mentioned their engagement to combat the pandemic. Among these, three of them had prepared standard alcohol-based hand-rub in collaboration with other bodies. Moreover, as we have checked on direct observation all of the premises had water tank with soap in the main gateway for hand wash and sanitizer, alcohol and were advising clients to keep physical distancing.

*“… We are applying all the prevention mechanisms WHO recommends. We have prepared alcohol-based sanitizer in collaboration with other bodies as the case is difficult to work and control alone. There is a taskforce organized from different professionals to control COVID-19, and we are actively working with the team especially in supplying sanitizers, drugs and medical supplies used as supportive treatment of the disease.” (C19Phar001)*

### Rational drug use and COVID-19

All study informants (*n* = 6) responded that there was some tendency of change in the management and rational use of drugs. There was shortage of personal protective equipment and other medical supplies in the market. During the beginning of the lockdown customers asked to procure antibiotics and anti-malarial drugs. This was strengthened by one informant that:

*“… There was a minor change in the drug supply system and rational drug use because of COVID-19. This also imposed misunderstanding in the population especially in the beginning, late February 2020. During that time, many of my customers had asked me for prescription drugs and medical supplies. However, nowadays the population seems calmed which might be after the regional health bureau and we professionals had created various awareness programs.” (C19Phar003)*

Another informant stated:

*“…After new confirmed case was reported in our country some clients asked to get Chloroquine and Azithromycin as they heard they can be cured if they get sick. But I have clarified to them these medications are prescription only drugs and are not approved for the treatment COVID-19.” (C19Phar006)*

### Customer wrong beliefs about COVID-19

All study informants (06) mentioned customers were with false claims in the beginning of the pandemic. They further responded; this might be because of usual traditional medication practice used commonly in the community.

*“… some customers asked me if home remedies like garlic can prevent the disease and I have responded them these home remedies might have their own health value but there is no approved product for treatment of COVID-19. I advised them to follow the instructions given by healthcare providers, regional and national health bureaus, and WHO for reliable information.” (C19Phar004)*

### Challenges due to COVID-19 period

Study informants were asked if they face problems due to COVID-19 while in practice and to rate the status the pharmaceutical care service delivered. All of them (*n* = 6) mentioned that their revenue has decreased due to the introduction of lockdown. Besides, they reported there is negligence in the population in applying the prevention strategies though they are informing the population to do so. However, although the economic income has decreased, they have rated the pharmacy service provided was very good.

*“… in this regard similar to the country economic recession my income is substantially decreased which might be because of quarantine instructed by the regional government. I hope this will be for the short period of time. The other challenging issue is peoples are not following the instructions given to prevent the disease. They are very negligent though we are counseling them to follow all the mechanisms to halt the spread of the disease. Although we are facing with these and other challenges, the pharmacy service is being delivered very well.” (C19Phar005)*

### Collaboration with other bodies

Collaboration with other stakeholders was considered as a must do protocol to combat COVID-19. All the interview informants (*n* = 6) have responded they were working with other healthcare professionals, COVID-19 prevention and control taskforce, clinics, hospitals, regional health bureau and other relevant stakeholders.

*“… this disease is difficult to control apart that is why I am working with the command post and clinics around, to report if I found any suspected case. I have the call number of the local government registered in my notebook which is assigned to inform and ask any of COVID-19 concern.” (C19Phar004)*

### Patient education and support

Our finding revealed, patient education and psychological support were being delivered. The strategies necessary outlined for the prevention and control of the pandemic were being given. Customers were counseled to stay at home, wash their hands or use hand sanitizer and maintain physical distancing.

*“… We are educating them to apply all the prevention mechanisms given by WHO, to exercise at home and out of home. We are instructing them to stay at home unless they have an urgent issue that necessities them to go out. If they go out of home, we recommend them to stay away from crowded areas, wash or sanitize their hand and use face mask as possible.” (C19Phar002)*

In addition, the case of COVID-19 has introduced some tendency of apprehension and psychological disturbance in the population. In our study, the study informants responded many of their customers have asked them what could be the fate of COVID-19.

*“… yes, some of my customers came and asked me what they should do and what will be the fate after the case. I have seen some degree of fear and I have tried to stabilize them… instructed them as the disease is not such a big threat… just we can control it if we stay in home, avoid crowded areas and physical contact until the pandemic is controlled and a solution is found.” (C19Phar006)*

## Discussion

To the best our knowledge this is the first study to assess and explore the preparedness of pharmacies and pharmacy professionals to prevent and control the pandemic disease COVID-19 in Ethiopia. The study found necessary preparedness measures were undertaken to control the current public health threat of COVID-19 regarding pharmacy service. In line with other studies recommendations [12, 13], community and hospital pharmacists were working at the frontline to combat the pandemic disease. This helps in the urgent control of public health emergencies like COVID-19.

The International Pharmaceutical Federation (FIP) and other studies recommend, in addition to the usual services provided, ensuring pertinent pharmacy service is required to prevent and control COVID-19. This includes ensuring adequate storage and supply of drugs and medical supplies, promoting rational drug use, point of care tests, and working in collaboration and reporting any suspected case to the responsible body [6, 8, 11, 13–15]. Similarly, the present study has revealed the pertinent pharmacy services were evidenced although supply of some drugs and medical supplies were scarce.

The tendency of customers to false claims of medical products and assumptions that helps to detect, prevent and control was showed especially in the introduction of the pandemic disease. The WHO medical product alert No. 3/2020 has warned health providers, responsible bodies, and customers to work against the production of falsified medical products [16]. Later on, customers were getting informed as there was no approved drug or vaccine for the treatment and prevention of the disease. Similarly, the FIP and Sandres et al reported no cure has been found to date [17, 18]. Although the American Food and Drug Administration has authorized an emergency drug Remdesiver which showed an effect against COVID-19 [19, 20], our finding implicates customers should adhere the counseling tips given by pharmacy professionals to keep themselves free from the pandemic disease.

Similar to our finding, public health emergencies like COVID-19 need a multidisciplinary effort to control and improve patients treatment outcome [12]. Due to the more accessible nature of community pharmacies, linking patients who are suspected to COVID-19 to healthcare system is feasible and more helpful to contain the pandemic disease [21, 22]. In addition; staff shifting, backup plans, alternative pharmacy services and other intervention mechanisms should be considered to halt the spread of the disease.

Patients presented with no symptoms of COVID-19 should be provided evidence based information and advice [23]. Population with different age group and disease classification might visit these pharmacies for service. All infection prevention and control measures should be considered meticulously including in healthcare settings [24, 25]. The study revealed, customers were learned to be calmed and get informed all the prevention strategies to cope with COVID-19 as some guidelines recommend [24, 26]. This eases the scrutinized and collaborative control of the pandemic disease. Similar to our finding, pharmacy professionals can found updated evidence about COVID-19 in various databases, web pages and social media outlets about COVID-19 [27].

As the present study disclosed, the population seems to be careless and loose in strictly following the instructions given to prevent and control the pandemic disease. This might be due to the country and worldwide economic recession and other factors imposed as of the lockdown. This imply the government and other responsible body to take a proactive measures to solve the problem [28, 29].

### Limitation of the study

Our study has limitations. Due to the nature of the study design and as the study was conducted in one of the large cities in Ethiopia but with limited number of pharmacies, the findings may not be generalizable. However, our finding would add to the growing literature particularly in the area of pharmacy service in public health emergencies and pharmacy preparedness to prevent and control the pandemic COVID-19 in Ethiopia, and other LMICs.

## Conclusion

The study revealed necessary pharmacy services were being given. However, sufficient supplies of drugs and medical supplies were scarce which negatively affected the optimal delivery of pharmacy services. Further, the tendency of following the prevention strategies against COVID-19 has been found loose in the population. The government and other responsible bodies should work together to solve such problems and contain the pandemic.

## Supplementary information

**Additional file 1.** Data collection instrument

## Data Availability

The datasets used and/or analyzed during the current study are available from the corresponding author on reasonable request.

## Abbreviations

COVID-19: Coronavirus Disease 2019
FIP: International Pharmaceutical Federation
FMoH: Federal Ministry of Health
LMICs: Low- and Middle-Income Countries
WHO: World Health Organization
