# Supplementary material for: Pharmacy preparedness and response for the prevention and control of coronavirus disease (COVID-19) in Aksum, Ethiopia; a qualitative exploration
Source: BMC Health Serv Res. 2020 Oct 2;20:913. doi: 10.1186/s12913-020-05763-9 (PMC7530845; doi:10.1186/s12913-020-05763-9)
Supplement: Supplementary file 1 — Additional file 1. Data collection instrument [file 12913_2020_5763_MOESM1_ESM.docx]

**Data collection instrument**

**Aksum University**

**College of Health Sciences**

**Department of Pharmacy**

**Semi-structured interview guide and observational checklist**

**Title**: Pharmacy Preparedness and Response for the Prevention and Control of Coronavirus Disease (COVID-19) in Aksum, Ethiopia; A Qualitative Exploration (May, 2020)

|  | **Part I: Identification** | | | |
| --- | --- | --- | --- | --- |
| Identification Code: __________ | | | Date: _____ | |
| Data collector name: ______________ | | | Sig:_______ | |
|  | **Part II: Socio-demographic characteristics (please answer the questions accordingly)** | | | |
| **S.no** | **Question/s** | **Response** | | |
|  | Age | _______ | | |
|  | Gender | 1. Male 2. Female | | |
|  | Qualification | 1. Pharmacist 2. Pharmacy technician 3. Other | | |
|  | Level of study | 1. Certificate 2. Degree 3. M.Sc. 4. Other | | |
|  | Years of experience | _______ | | |
|  | **Part III: Awareness and preparedness against COVID-19** | | | |
|  | What is COVID-19?  (Probing: what is COVID-19, transmission mechanisms, and prevention strategies?) |  | | |
|  | What did you refer to get the evidence about COVID-19? |  | | |
|  | What measures are you taking in combating COVID-19?  (Probing: hand washing, preparing/sanitizer application …others) |  | | |
|  | How is the rational use of medicines and its dispensing practice during COVID-19? |  | | |
|  | What do you think the drug supply status for the treatment of COVID-19? |  | | |
|  | How is the general stock status you have? |  | | |
|  | How is the risk of hoarding medicines and medical supplies? |  | | |
|  | What do you say about false claims, false vaccines and drug misinformation/practice?  How do you prevent for such cases? |  | | |
|  | What problems are you facing due to the pandemic? |  | | |
|  | **Part IV: Patient education measures given** | | | |
|  | What did you counsel patients/clients to do in home and/or out of home for the prevention of COVID-19 transmission? |  | | |
|  | What did you counsel for patients with anxiety/fear because of COVID-19?  (Any psychological support?) |  | | |
|  | What strategies could you use in combating COVID-19?  (Probing: Collaboration with local and national public health authorities and other relevant agencies) |  | | |
|  | How do you rate the access of pharmaceutical care during COVID-19? (please encircle) | 1. Very good 2. Moderate 3. As usual  4. Not satisfactory 5. Not very satisfactory | | |
|  | **Part V: Self-initiated preparedness measures taken (please encircle)** | | | |
|  | Soap and water in-front of the medicine outlet | | | 1. Yes 2. No |
|  | Alcohol/Hand rub around | | | 1. Yes 2. No |
|  | Clients getting in and out 2m apart | | | 1. Yes 2. No |
|  | Compounding of sanitizer or availing any WHO recommended ABHR | | | 1. Yes 2. No |
|  | Creating awareness for customers about COVID-19 and its prevention mechanisms | | | 1. Yes 2. No |
|  | Informing clients to report any suspect to relevant bodies | | | 1. Yes 2. No |
|  | Collaboration with local and nation agencies (contact no, address available) | | | 1. Yes 2. No |
|  | Staff training | | | 1. Yes 2. No |
